# A Novel Ruthenium Based Coordination Compound Against Pathogenic Bacteria

**DOI:** 10.3390/ijms21072656

**Published:** 2020-04-10

**Authors:** Vishma Pratap Sur, Aninda Mazumdar, Pavel Kopel, Soumajit Mukherjee, Petr Vítek, Hana Michalkova, Markéta Vaculovičová, Amitava Moulick

**Affiliations:** 1 Department of Chemistry and Biochemistry, Faculty of AgriSciences, Mendel University in Brno, CZ-613 00 Brno, Czech Republic; xmazumda@mendelu.cz (A.M.); xmukher1@mendelu.cz (S.M.); hanabuchtelova@gmail.com (H.M.); marketa.ryvolova@seznam.cz (M.V.); 2 Central European Institute of Technology, Brno University of Technology, CZ-61200 Brno, Czech Republic; 3 Department of Inorganic Chemistry, Faculty of Science, Palacky University, CZ-771 46 Olomouc, Czech Republic; paulko@centrum.cz; 4 Global Change Research Institute of the Czech Academy of Sciences, CZ- 603 00 Brno, Czech Republic; vitek.p@czechglobe.cz

**Keywords:** coordination compound, antimicrobial compound, ruthenium, benzimidazole, SEM, EDS

## Abstract

The current epidemic of antibiotic-resistant infections urges to develop alternatives to less-effective antibiotics. To assess anti-bacterial potential, a novel coordinate compound (RU-S4) was synthesized using ruthenium-Schiff base-benzimidazole ligand, where ruthenium chloride was used as the central atom. RU-S4 was characterized by scanning electron microscope (SEM), energy-dispersive X-ray spectroscopy (EDS), and Raman spectroscopy. Antibacterial effect of RU-S4 was studied against *Staphylococcus aureus* (NCTC 8511), vancomycin-resistant *Staphylococcus aureus* (VRSA) (CCM 1767), methicillin-resistant *Staphylococcus aureus* (MRSA) (ST239: SCCmecIIIA), and hospital isolate *Staphylococcus epidermidis*. The antibacterial activity of RU-S4 was checked by growth curve analysis and the outcome was supported by optical microscopy imaging and fluorescence LIVE/DEAD cell imaging. In vivo (balb/c mice) infection model prepared with VRSA (CCM 1767) and treated with RU-S4. In our experimental conditions, all infected mice were cured. The interaction of coordination compound with bacterial cells were further confirmed by cryo-scanning electron microscope (Cryo-SEM). RU-S4 was completely non-toxic against mammalian cells and in mice and subsequently treated with synthesized RU-S4.

## 1. Introduction

The appearance of antibiotic-resistant pathogenic bacteria during mid-nineteenth century created an urge to develop a treatment against these life-threatening pathogens, alongside the existing drug molecules known as antibiotic. The present scenario in terms of the effectivity of novel antimicrobials is quite complex due to the rapid uprising of antibiotic-resistance in bacteria. Their ability to evade the effect of antibiotics by using various mechanisms depends on their evolving nature, which makes these pathogens smarter and better. Antibiotic resistance is an emerging problem in society and a crucial challenge to medical biology. The epidemiology of *Staphylococcus aureus* and the antibiotic-resistant *S. aureus* strains are changing constantly. *S. aureus* causes a range of mild to life-threatening community-associated infections and currently is more significant with the evolution of antibiotic-resistant species like vancomycin-resistant *S. aureus* (VRSA), and, methicillin-resistant *S. aureus* (MRSA) [1]. VRSA and MRSA are some of the primary causes of nosocomial infections associated commonly with increased morbidity and increases treatment duration and medical cost [2]. The VRSA strain is thought to acquire the vancomycin-resistance gene from the antibiotic-resistant vancomycin-resistant Enterococci (VRE), another predominantly concerning pathogen that causes infections similar to VRSA and MRSA [3]. Initially, penicillin used to be the major effective treatment for *S. aureus* infections until the efficacy of the antibiotic reduced. It is due to notable evidence of acquiring the penicillin-resistance gene and consequently the development of resistant mechanism toward prominent broad-range antibiotics, such as methicillin and vancomycin [4].

Contrary to efficacy, both incomplete dosage and over-usage of antibiotics cause side effects which further complicates the well-being of the patient. The use of broad-range antibiotics such as vancomycin was considered as one of the last resorts against significantly life-threatening, multidrug-resistant infections caused by gram-positive bacteria [5,6]. However, the increasing complexity of different antibiotic resistance mechanisms in *S. aureus* strains such as VRSA and MRSA resulted in a significant negative impact on health and clinical settings. With the increasing mortality rate causing by antibiotic-resistant bacterial strains and with fewer appropriate treatment options for multi-drug resistant infections, currently, there is a necessity to investigate new candidates as potential alternative to antibiotics. In pursuit of new effective and non-cytotoxic antimicrobials, recent reports witnessed a rise in the usage of novel metal-based coordination compounds, nanoparticles (NPs) [7,8,9,10,11] or antimicrobial peptides as alternatives to existing antibiotics. It has been previously reported that transition metal-based coordination compounds and ligand-based coordination compounds were used as potential antimicrobial agents [7,9,10,11,12].

Thus, in this study, we have aimed to develop a novel metal-based coordination compound to treat life-threatening infections caused by *S. aureus*, VRSA, MRSA, and *Staphylococcus epidermidis* (isolated and obtained from hospital sample). In this study ruthenium-based coordination compound synthesized with benzimidazole and Schiff base ligands (ruthenium-Schiff base–benzimidazole coordination compound). Benzimidazole derivatives are heterocyclic molecules that have a wide range of biological activities and reported to have antibacterial, antimicrobial, anthelmintic, analgesic, antitumor, and anti-inflammatory properties [7,13]. In other studies, it has been shown to inhibit DNA gyrase and to exhibit DNA binding affinity [14,15]. Thus, benzimidazole derivatives can be used as the potential candidates for developing new biologically active compounds [14]. Schiff bases are versatile biologically active molecules with antimicrobial activity and being used for drug molecule designing and industrial purposes [12]. Ruthenium-based compounds are well-known antibacterial compounds, where ruthenium played a key role [9,10,11]. Whereas, ruthenium provides the metal ion background and the complexes formed by them have biological importance. Thus, the ruthenium-Schiff base benzimidazole coordination complex with a unique combination was synthesized as new novel compounds to study its antimicrobial efficacy.

Further, the chemical characterization of ruthenium, Schiff base and benzimidazole were carried out using biophysical techniques like Fourier-transform infrared spectroscopy, scanning electron microscope and Raman microscopy. Energy-dispersive X-ray spectroscopy (EDS) elemental analyses were performed to confirm the presence of all the individual components. Further, the effect of this ruthenium-based coordination compound was used to study the antibacterial efficacy against *S. aureus*, VRSA MRSA, and *S. epidermidis*. Thereafter, the MIC was calculated and the lowest concentration with the highest effectivity against bacterial growth was used for the toxicity test. The toxicity test was performed by several assays including MTT assay and hemolytic assay. In vivo study of the RU-S4 was also carried out to better understand its biocompatibility and activity towards *S. aureus* and its resistant strains.

## 2. Results

### 2.1. Chemical Synthesis and Electron Microscopy and Energy-Dispersive X-ray Spectroscopy (EDS) Confirmation

**Figure 1:**
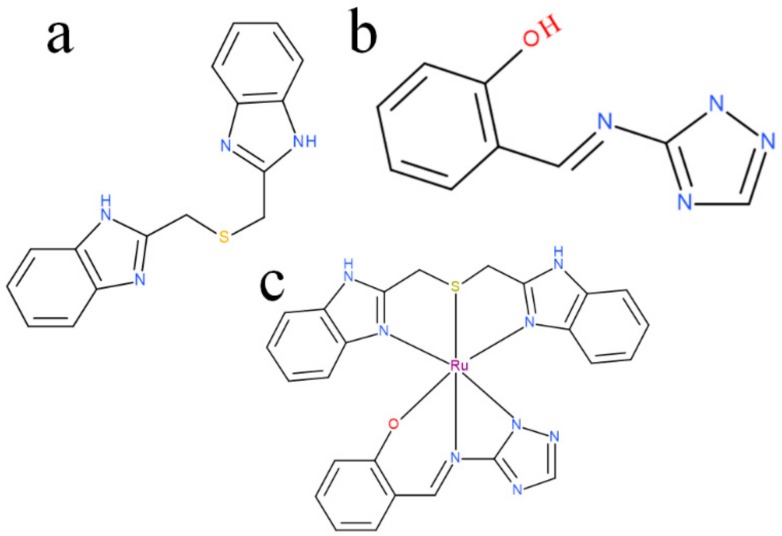
(**a**) benzimidazole ligand structure, (**b**) Schiff base ligand structure, (**c**) possible structure of RU-S4.

**Figure 2:**
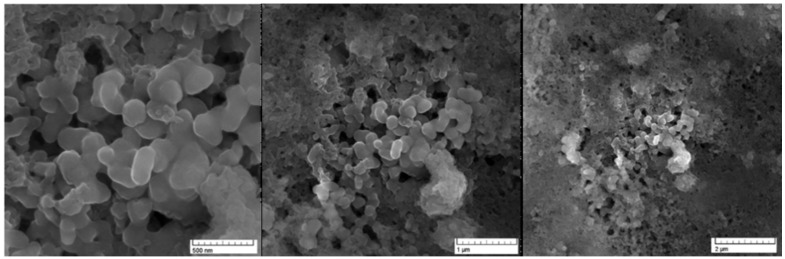
Scanning electron microscopy image for RU-S4 in different scales: (**a**) scale 500 nm, (**b**) scale 1 µm, (**c**) scale 2 µm.

The ruthenium-Schiff base–benzimidazole complex (chemical structure of benzimidazole ligand, Schiff base ligand and probable structure of RU-S4, Figure 1) and the Energy-dispersive X-ray spectroscopy (EDS) method confirms that in the composed complex have 39% (±2) ruthenium (Figure S2). The presence of nitrogen 41% (±2) proves that this compound contains nitrogenous benzimidazole and Schiff base and ruthenium (Figures S1 and S2). Visualization of the prepared complex on scanning electron microscope (SEM) shows partial particle character of the sample. The average diameter of the greater particles is about 500 nm, but the smaller entities have a size of about 100 nm and less. The formation of bigger particles was assumed as a result of sample drying for SEM visualization. In solution, we suppose the average size of entities is about 100 nm (Figure 2).

### 2.2. Characterization of the Ru Complexes by Raman Spectroscopy

**Figure 3:**
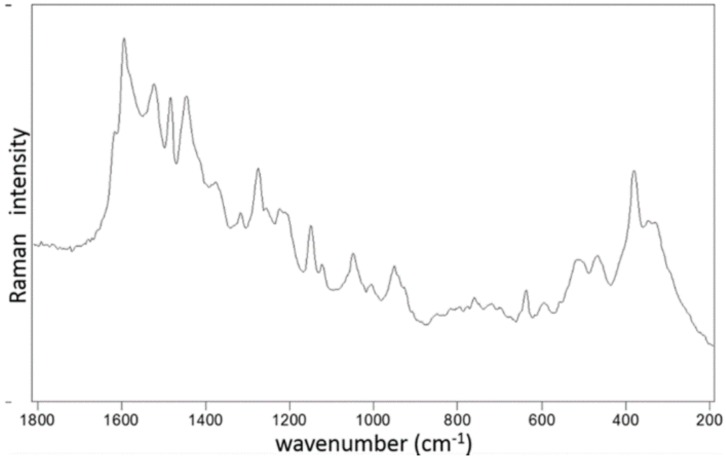
Raman spectra and listed bands of Ru-S4 complex as obtained by the 514.5 nm excitation.

Raman spectra of Ru-S4 complex provide a spectral pattern, with bands assignable to benzimidazole located at 1006, 1048, 1276, 1318, and 1378 cm^−1^ (Wang et al., 2004, Suwaiyan et al., 1990) [16,17]. Features attributed to Schiff base are located at 1582 and 1618 cm^−1^ (Figure 3).

### 2.3. Growth Curve Snalysis

**Figure 4:**
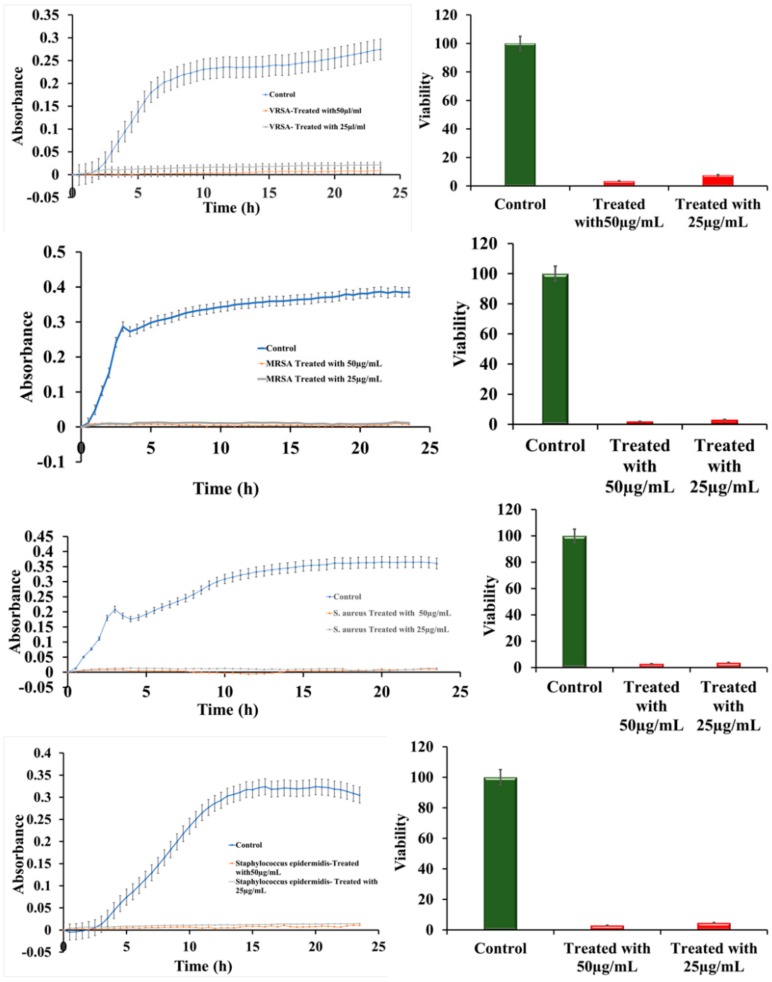
Bacterial growth curve and viability curve (**a**–**d**); The growth curve of VRSA (CCM 1767), MRSA (ST239: SCCmecIIIA), *S. aureus* (NCTC 8511), *Staphylococcus epidermidis* respectively (**e**–**h**); Viability percentage of VRSA (CCM 1767), MRSA (ST239: SCCmecIIIA), *S. aureus* (NCTC 8511), hospital sample *Staphylococcus epidermidis* respectively. Data represent the mean ± SD, *n* = 3.

The effect of ruthenium-Schiff base−benzimidazole (Ru-S4) complex compound was investigated against VRSA (CCM 1767), MRSA (ST239: SCCmecIIIA), and the hospital sample *Staphylococcus epidermidis* also. The effect of the RU-S4 compound and the MIC were determined by bacterial growth curve analysis [18]. Antibacterial activity of the RU-S4 compound was studied by turbidimetry technique, the absorbance was measured at 600 nm for 24 h against MRSA (ST239: SCCmecIIIA), VRSA (CCM 1767), *S. aureus* (NCTC 8511), and hospital sample *Staphylococcus epidermidis* [19,20,21,22,23,24]. The bacterial viability was calculated as mentioned in our previous work [25]. It was evaluated from the growth curve and viability curve that the ruthenium compound has the most adverse effects on all bacterial species used in this experiment. Different concentration was used for application, but 25 µl/mL and 50 µl/mL concentration show most adverse effects against all bacterial populations in both commercial bacteria and hospital samples also. For all bacterial populations MIC value was 25 µg/mL (except 25 µg/mL and 50 µg/mL no other concentrations showed relevance effect) Figure 4a–d. The percentage of viability for VRSA (CCM 1767) resulted > 8 and > 4 with a concentration of Ru-S4 of 25 μg/mL and 50 μg/mL, respectively. The percentage of viability for MRSA (ST239: SCCmecIIIA) was > 4% with a concentration of Ru-S4 of 25 μg/mL and > 2% with a concentration of 50 μg/mL. The percentage of viability for (NCTC 8511) resulted > 4 and > 3 with a concentration of Ru-S4 of 25 μg/mL and 50 μg/mL respectively. The percentage of viability for *S. epidermidis* was > 3% with a concentration of Ru-S4 of 25 μg/mL and > 5% with a concentration of 50 μg/mL 

**Figure 5:**
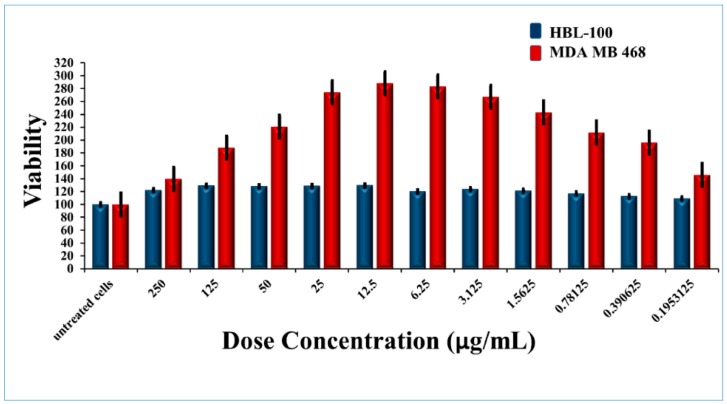
Cytotoxicity for RU-S4 against the human cell line.

**Figure 6:**
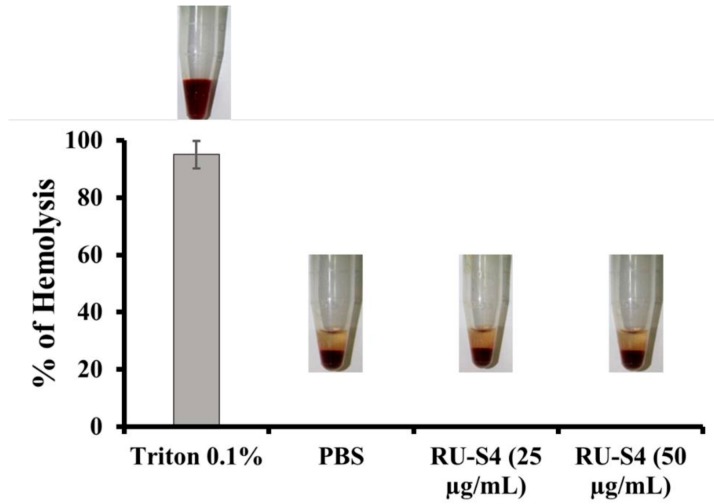
Percentage of hemolysis of blood cells treated with RU-S4 in a blood sample.

The cytotoxicity of Ru-S4 was estimated by using normal breast cell line HBL 100 and Cancer Breast cell line MDA MB-468 and prostate epithelial cell line PNT1A. RU-S4 showed negligible toxicity towards the all cell lines (Figure 5, Figure 6, Figure S9). The end conclusion is RU-S4 is completely nontoxic.

### 2.4. Hemolytic Assay

Drug-induced hemolysis is a major toxicity problem. High drug concentrations can also be a cause of hemolysis inpatient. US FDA recommended hemolysis screening for drug molecules is a very important requirement of drug development [26].

It is clear from this Figure 6 that RU-S4 exhibited no hemolysis, where 0.1% TritonX exhibits 100% hemolysis.

### 2.5. Optical Microscopy

**Figure 7:**
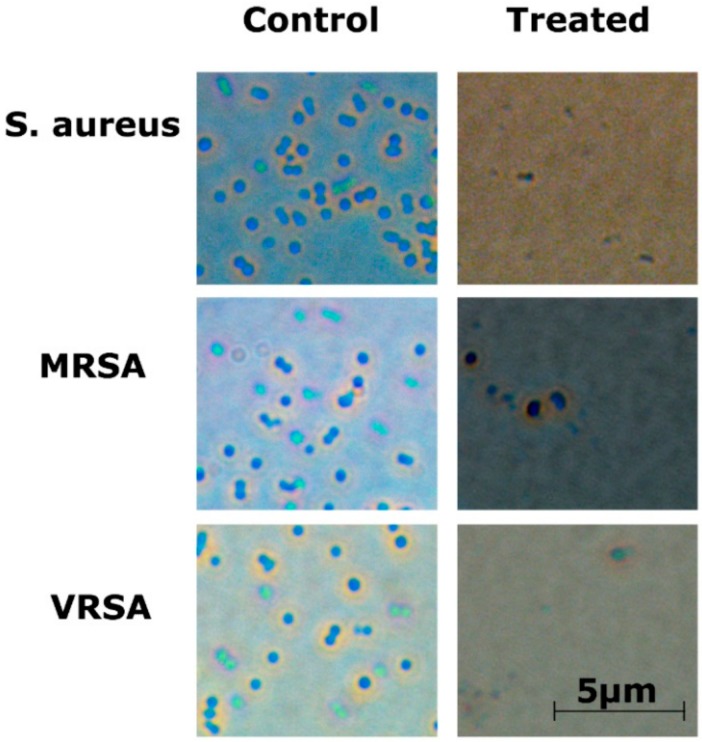
Optical microscopy image for RU-S4 treatment against *S. aureus* (NCTC 8511), MRSA (ST239: SCCmecIIIA), VRSA (CCM 1767), and untreated cells. No morphological changes were seen in untreated cells whereas the treated bacterial cells were ruptured and almost no visible structured cells can be seen. Scale bar is 5 µm.

Optical microscopy showed that bacterial cells were disrupted after treatment with the RU-S4 complex (Figure 7 and Figure S13).

### 2.6. LIVE/DEAD Cell Imaging by Fluorescence Microscopy

**Figure 8:**
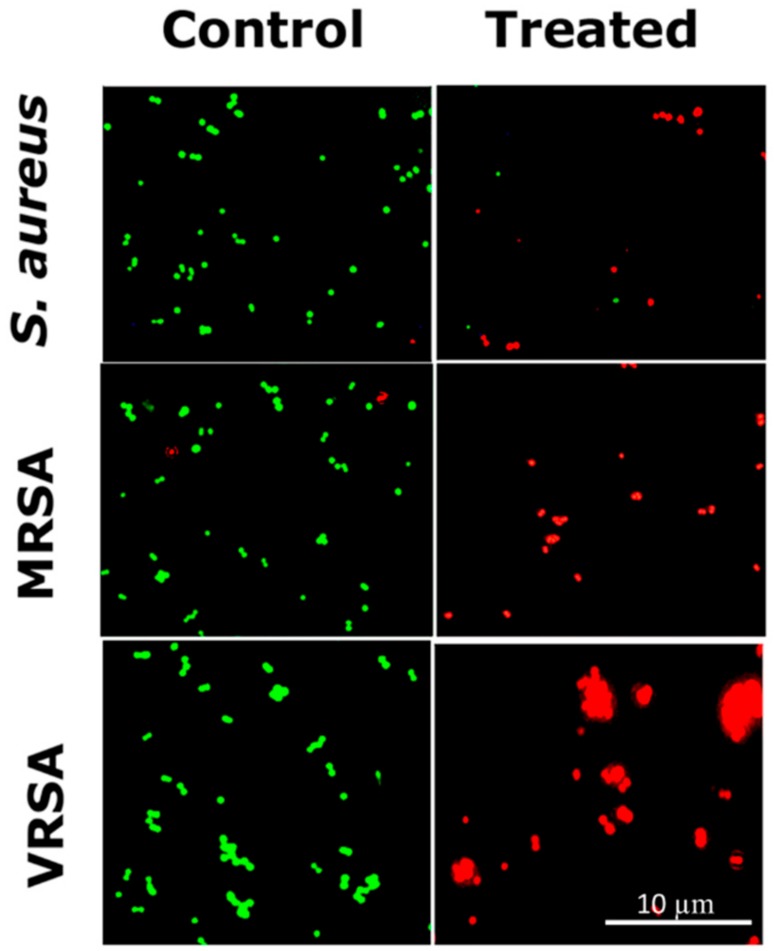
LIVE/DEAD cell imaging for RU-S4 treatment against *S. aureus* (NCTC 8511), MRSA (ST239: SCCmecIIIA), and VRSA (CCM 1767). Green cells define living cells whether red cells stand for dead cells. Scale bar is 10 µm.

The viability of *S. aureus*, VRSA (CCM 1767), and MRSA (ST239: SCCmecIIIA) (Figure 8) and *S. epidermidis* (Figure S14) after treatment with RU-S4 was observed by the LIVE/DEAD cell imaging assay [8,25,27]. The bacterial samples treated with RU-S4 showed considerably decreased cell count with green fluorescent, but a significant increase in number of dead cells with red fluorescence, whereas in the case of control bacterial cells, the number of green fluorescent cells were high with red fluorescence (Figure 8 and Figure S14).

### 2.7. Cryo-SEM Microscopy Imaging for Bacterial Cells Treatment with RU-S4

**Figure 9:**
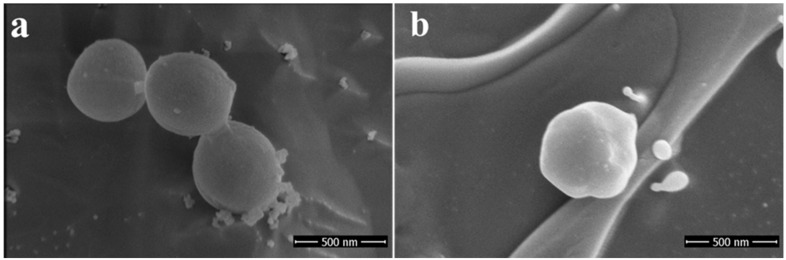
Scanning electron microscope (SEM) image: (**a**) left panel shows untreated coccus cells (VRSA) (CCM 1767), (**b**) right panel shows treated coccus (VRSA) (CCM 1767).

VRSA (CCM 1767) cells treated with RU-S4 and imaged under scanning electron microscope, where untreated bacterial cells appeared as intact cocci shape with no cell wall rupture or collapse (Figure 9). On the other hand, bacterial cells treated with RU-S4 showed cell outer shape bubbling rough surface and clumped and deformed which can be seen in Figure 9 [28].

### 2.8. In Vivo Animal Model

**Figure 10:**
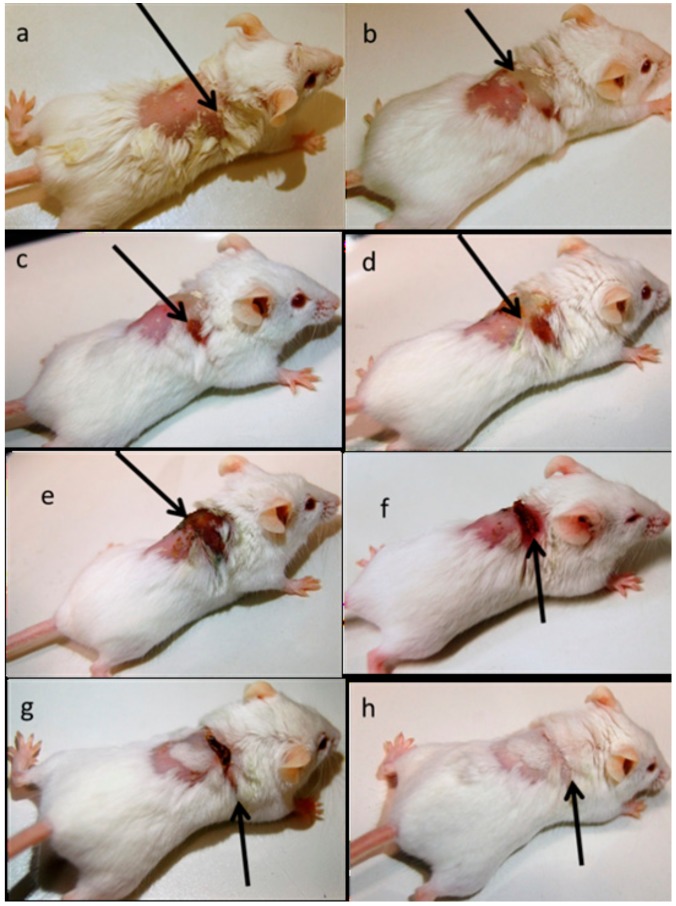
In vivo infection model preparation, treatment, and recovery: (**a**) after bacterial infection dose, (**b**) day 1 Infection initiation, (**c**) day 2 Infection and wound growth, (**d**) day 3 inflammation and swelling, (**e**) day 6 no significant changes were observed, (**f**) day 9 wound started to heal, (**g**) day 12 wound started to heal, (**h**) day 15 fully recovered.

After the infection started spreading in the mouse near the dermal part near neck swells and wound started to form. After swelling and wound formation their treatment was started with RU-S4 and day by day infection and wound started to heal and their skin started to recover from the traumatic infection situation which can be seen in Figure 10. Here we use uninfected control (Figure S10). The control animal was maintained throughout the experiment for monitoring, wherever no changes were found. Whereas infected untreated control animals were monitored (Figure S11). During the experimental condition the morbidity of the infected untreated animals were 100% within 8 days, where dermal infection growth and simultaneously internal infection growth were observed.

**Figure 11:**
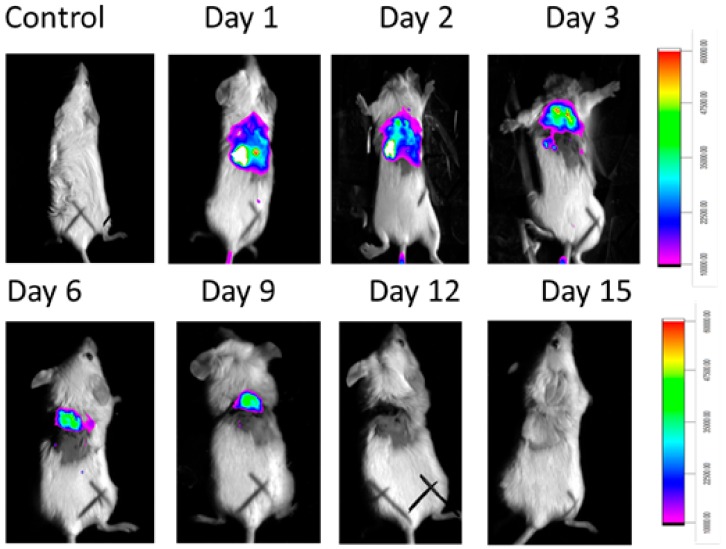
In vivo experiment where MMP Sense fluoresces in infected mice not in control and with time intervals the fluorescence disappears due to the recovery.

MMPSense was also gave fluorescence (against infection) until 9 days. From day 12 of observation there was no fluorescence for MMPSense (Figure 11). The decreased MMP (Matrix metallopeptidases) fluorescence intensity was also decreased (Figure S12).

## 3. Discussion

EDS analysis of the RU-S4 complex confirms the uniform distribution of individual elements (Figures S1 and S2). This resembles the morphology of the compound and thus confirm the homogeneity of the prepared complex. SEM images of the compound shows spherical shape and holds the structure in solution (Figure 2). X-ray analysis was performed with the complex without good outcomes due to the low quality of crystals. Whereas Raman spectrum data confirms the presence of ruthenium, Schiff base, and benzimidazole base ligand (Figure 3).

Growth curve analysis shows bacterial cell viability near about 2–4% when treated with RU-S4 Figure 4e–h). According to previous reports, ruthenium, ruthenium-Schiff base and benzimidazole individually shows strong activity against antibiotic-resistant strains and also to wild types [29,30,31]. According to the literature review, no compound was synthesized combining all these three elements and reported to show antibacterial action against VRSA (CCM 1767), and MRSA (ST239: SCCmecIIIA), *S. aureus* (NCTC 8511), and *S. epidermidis* (hospital sample) before this study. Based on our experiments, we found that the RU-S4 complex was able to kill almost 96–97% of bacterial populations (Figure 4a–d). Ruthenium with triazole derivative was reported to be effective against bacterial strains [32,33]. Previously the MIC value for the ruthenium was reported to be 128 µg/mL, whereas in our study the MIC value of RU-S4 was 25 µg/mL, which suggests its strong effect against bacterial population (Figure 4a–d). The mechanism for the antibacterial activity of ruthenium is not fully understood yet, whereas a few studies point to its ability to bind DNA and RNA or to its ability to generate reactive oxygen species effect [31,33,34,35]. This mechanism for strong bacterial elimination requires further study which we look forward to answering in our future research.

Ruthenium chloride, benzimidazole (2-(1H-benzimidazol-2-ylmethylsulfanylmethyl)-1H-benzimidazole) and Schiff base (2-((E)-1H-1,2,4-triazol-5-yliminomethyl) phenol), were applied individually against bacterial samples and no significant changes were observed (Figures S3–S8). Even after the addition of compounds mixture (1:1:1 ratio) to bacterial sample, no significant changes were observed. Whereas our novel RU-S4 complex exhibited high antibacterial activity and we suggest it as a novel alternative to antibiotics to treat drug-resistant bacteria.

Cytotoxicity assay and hemolytic assay confirmed that the synthesized compound RU-S4 is almost non-toxic for mammalian cells and it can be further formulated for treatment (Figure 5, Figure 6 and Figure S9). The MTT assay reflects metabolic healthy conditions of cells by estimating mitochondrial succinate dehydrogenase. Mitochondrial succinate dehydrogenase reduces MTT and water-insoluble purple color formazan crystal was formed, and the amount of formazan produced is directly proportional to viable cells [25,26]. From the graph, we can conclude that after the application of all desired concentrations of RU-S4 no significant cell mortality rate was found (Figure 5). So, from this point of view and our graph analysis, we can say that RU-S4 is a nontoxic compound because a high number of cells mean more formazan produced by mitochondrial succinate dehydrogenase which proves that all cells were highly metabolically active.

In Vitro hemolysis result analysis of RU-S4 was carried out at 25 µg/mL and 50 µg/mL concentration as shown in Figure 6. From this experiment it is clear that the RU-S4 exhibited no hemolysis. where 0.1% TritonX exhibits 100% hemolysis (Figure 6), and it can be concluded that RU-S4 exhibits no toxicity and it can be use as potential molecules for further drug formulation.

The optical microscopic image is showing bacterial cell debris, which supports that our compound (RU-S4) have antibacterial activities. This microscopic image strongly supports that the RU-S4 complex compound disrupts the cell membrane also (Figure 7 and Figure S13). This fluorescence microscopy results showed a prominent reduction in the bacterial viability, treated with RU-S4. The reduction of bacterial cell death by cell wall disruption, cell morphology change, and cell death, which can conclude from optical microscopy (Figure 7) and fluorescence microscopy images (Figure 8). This result further support that synthesized RU-S4 has high antimicrobial activity [25,36]. Optical microscopy along with fluorescence LIVE/DEAD cell imaging and SEM imaging shows bacterial cells are damaged, deformed, and lysed (Figure 7 and Figure 8). The Cryo-SEM results show loss of bacterial cell wall integrity and change in morphology, cell wall breakage (clear from the Figure 9), possibly due to stress and underlies the possible disruption of the bacterial cell wall by RU-S4 [25,28,36].

Finally, the in vivo study shows the biologically active RU-S4 has high antibacterial activity (Figure 10 and Figure 11). Infected mice treated with a very low concentration of RU-S4 were completely cured. Whereas infected mice without drug administered went through dermal and internal growing infection, fever, and whole-body twitching. One of the most prominent findings from this study was the complete cure of bacterial infection in mice by combination of subcutaneous injection and spraying the RU-S4 compound, which opens an opportunity to use RU-S4 as an antibacterial drug in aerogel/hydrogels as in ointments. Although in vivo fluorescence imaging (Figure 11) supports the pictorial conclusion. This imaging was done by MMPSense which is optically silent but activated after cleaved by matrix metalloproteinase (MMPs). MMPs are the family of enzymes that are present almost in all inflamed human tissues, however, they play role in mice also [37]. According to Caley et.al. MMP knocked out mice shows very slow wound healing capacity [38]. In this experiment normal balb/c mice were taken, and it is normal that after infection and wound formation, MMPs were secreted [38] and cleave our MMPSense 645, which gave fluorescence signals through imaging platform. Figure 10 suggests that after starting treatment with RU-S4 day by day fluorescence intensity was getting lower and from this observation (Figure S12), it is clear that MMPs secretion was also getting lower and all treated mouse started to heal Figure 11. This reference is to represent normal optical imaging of mice during infection growth and treatment start [39]. MMP Sense signaling imaging supports that RU-S4 has very high antibacterial activity [40]. MMP Sense fluorescence intensity also decreases day by day due to the cure of animal infection wound and less MMP and less amount of cleaving, so less fluorescence intensity Figure 11. The LD_50_ test confirms its toxicity level, the toxicity concentration was higher than the applied dose concentration (Supplementary experimental Section 1, Tables S1–S3).

To summarize, benzimidazole and Schiff base ligand-based ruthenium coordination compound was synthesized and the successful in vivo experiments on infected mice results in a complete cure of mice from VRSA (CCM 1767) without toxic effects toward mammalian cells. The mechanism, effectivity, and efficacy of RU-S4 require further study and this compound shows the potential to be a powerful alternative to antibiotics to treat multidrug-resistant *S. aureus* (NCTC 8511) infections.

Furthermore, the exact mechanism of first-time synthesized RU-S4 is not understood yet. However, the mode of action of antimicrobial agents may involve various targets in a microorganism. The possible mechanism is cell wall synthesis blockage and damage as a result of which cell permeability may be altered or may cell wall components like lipoprotein disorganize which can lead to cell death, or may various cellular enzymes deactivation and cellular biochemical or metabolic pathways interruption can lead cell death. Another hypothesis exerts that the cellular protein or proteins denaturation may interrupt and cellular cycle interruption and hydrogen bond formation through the azomethine group with the active center of cell constituents, which can start interference with the normal cell process [41]. Some studies suggest that transition metal complexes can bind to DNA via both covalent and non-covalent interactions [42]. The previous study also suggests that ruthenium complexes can interact with DNA, RNA, and protein as well [10,43]. Some studies suggest the metal center of the ruthenium binds to the biological target and the metal center of the coordination compound possess antimicrobial activity with biological targets [44], or the metal center of coordination compound which acts as active ligands carrier, normally-established drugs, to enhance their pharmaceutical activities via temporary coordination with the metal moiety [45]. Benzimidazole also has high DNA binding affinity [46]. From the previous studies, we can hypothesize that Schiff base interacts with the bacterial cell wall and make a path for the compound internalization and then the ruthenium core and benzimidazole both act with DNA, or block any enzymatic activity as well. However, the synthesized RU-S4 combination is completely new so extensive study on the antibacterial mechanism of RU-S4 is necessary. In our future perspectives, we are going to formulate aerogel trapped RU-S4 ointment.

## 4. Materials and Methods

### 4.1. Synthesis of Ruthenium-Schiff Base-Benzimidazole Complex (RU-S4)

All the chemicals were obtained from Sigma-Aldrich (St. Louis, MO, USA), with ACS purity unless noted otherwise. The ruthenium complex was synthesized by using a convenient co-ordinate complex compound synthesis strategy. First, benzimidazole based ligand 2-(1H-benzimidazol-2-ylmethylsulfanylmethyl)-1H-benzimidazole was synthesized according to our previous study [7]. During Schiff base (2-((E)-1H-1,2,4-triazol-5-yliminomethyl) phenol) synthesis 3-amino-1,2,4-triazole (1.68 g) and salicylaldehyde (2.13 mL) were heated under reflux in methanol (35 mL) for 32 h. During the time the color turned to yellow. After cooling to room temperature, yellow product was obtained and dried at 40 °C. Yield: 3.7 g. Anal. Calcd. C, 57.4; H, 4.3; N, 29.8. Found: C, 57.2; H, 4.3; N, 29.5%. Ruthenium chloride was dissolved in 20 mL of MeOH. Benzimidazole dissolved in the same solvent (5 mL) was added with stirring and the henceforth brown precipitate was formed. Afterward, the Schiff base was added (in MeOH 5 mL) followed by triethylamine. Brown solution with dark solids was heated under reflux for 48 h. After cooling, black solid was collected on a frit, washed with MeOH, and dried at 40 °C.

### 4.2. Electron Microscopy Imaging and Energy-dispersive X-ray Spectroscopy (EDS)

The morphology of the prepared material was studied using scanning electron microscope MIRA3 LMU (Tescan, a.s., Brno, Czech Republic). As an accelerating voltage of 15 kV and a beam current of about 1 nA were used for visualization with satisfactory results.

### 4.3. Raman Spectroscopy

Ruthenium complex was characterized by Raman spectroscopy, using a dispersive multichannel Raman microspectrometer InVia (Renishaw, Wotton-under-Edge, UK). The instrument is equipped with a confocal microscope (Leica, Wetzlar, Germany). For each measurement, a 50× magnification objective and Ar laser line at 514.5 nm were employed with 1 mW source power and 10 s exposure time accumulated 20× times. Raman spectra in the range of 250–2100 cm^−1^ were recorded. Spectra were baseline-corrected and smoothened (Savitzky-Golay) in WiRE 3.4 software (Renishaw, Wotton-under-Edge, UK) [16,17].

### 4.4. Bacterial cultures

Standard isolates of *S. aureus* (NCTC 8511), VRSA (CCM 1767), MRSA (ST239: SCCmecIIIA), hospital sample (*Staphylococcus epidermidis*) was used in this study. The used strains are either acquired from ATCC (USA) or Czech collections of microorganisms, Brno, Czech Republic (CCM). In this study, clinical sample Staphylococcus epidermidis (swab collected from hospital patient) was used. All bacterial isolates were grown in Mueller Hinton broth (Himedia laboratories Pvt. Ltd., Thane, India) in a shaking incubator at 37 °C. The composition of the growth media of the bacterial strains was as follows: Muller Hinton broth 21 g/L and the pH was adjusted to 7.4 [25].

### 4.5. Hospital Samples

Swabs collected from the bacterial infection place from the patients. The ethics committee of Trauma hospital in Brno, Czech Republic, approved this study according to act no. 378/2007 coll. The collection took place in trauma hospital, Brno. Swabs collected from infected wounds of patients and cultivated as mentioned earlier [16]. Bacterial strains were identified by 16S rRNA analysis capillary Sanger sequencing platform. Bacterial DNA was extracted by the NucleoSpin Microbial DNA kit (Machery-Nagel, Duren, Germany). The 16S rRNA gene fragments amplified by primer 8F and 1492R [17]. All obtained sequences were checked against NCBI database BLASTN. The antibacterial activity of RU-S4 was tested against bacterial sample isolated from hospital swab collection. The bacterial isolate was grown in Mueller Hinton broth (Himedia laboratories, Thane, India) in a shaking incubator at 37 °C. 

### 4.6. Bactericidal Effect of Ru-S4 and Growth Curve Analysis

The antibacterial activity of RU-S4 against different staphylococcal cultures, namely VRSA (CCM 1767) MRSA (ST239: SCCmecIIIA), *S. aureus* (NCTC 8511), and hospital bacterial samples collected from patients (*S. epidermidis*) was determined by the standard broth microdilution method and detected by quantitative growth curve analysis. RU-S4 solutions were added in concentrations of 25 µg/mL and 50 µg/mL into the microplate wells and were mixed with bacterial cultures (0.5 McFarland and final dilution 1:100 with the MH medium). The microplates with antimicrobial agents and bacterial cultures were incubated at 37 °C for 24 h. During incubation, the optical density of samples was measured by Bioscreen C MBR (Oy Growth Curves Ab Ltd, Vuorimiehenkatu 13, 00140 Helsinki, Finland) in every 30 min interval at 600 nm followed by 200 rpm shaking. The MIC was defined as the lowest concentration of antimicrobial agent that inhibited bacterial growth. The well with the concentration of lower dilution with no bacterial growth was considered the MIC. As a positive control, the MH medium with inoculation was used [18]. Effects of all individual components of the complex compound RU-S4 (ruthenium chloride, Schiff base, benzimidazole) were also checked on bacterial samples followed by the above-mentioned process.

### 4.7. In Vitro Cytotoxicity Assay

MTT assay is widely used for cell viability measurement, and it is dependent on mitochondrial respiration. To check cell viability, HBL 100 normal breast cell line and MDA MB 468 Breast cancer cell lines were established in RPMI media—1640 medium with 10% fetal bovine serum, supplemented with penicillin (100 mg/mL) and streptomycin (0.1 mg/mL). Microtiter plates were seeded with HBL-100 and MDA-468 separately and supplemented with 200 µL medium and incubated for 2 days at 37 °C. RU-S4 compound in higher to a lower amount (250 µL, 125 µL, 50 µL, 25 µL, 12.5 µL, 6.25 µL, 3.125 µL, 1.5625 µL, 0.78125 µL, 0.1953125 µL) was added to cells when the confluency of the cells reached—60%–80% cells. 24 h post-incubation old media was removed and 200 µL fresh media mixed with MTT (50 µL; 5 mg/mL in PBS) and incubated for 4 h at 37 °C in a humidified atmosphere followed by aluminum foil wrapping. After the incubation period MTT containing medium was replaced and 200 µL of 99.9% dimethyl sulphoxide (*v*/*v*) added for dissolving the formazan crystals. 25 µL of glycine buffer pH adjusted at 10.5 was added before taking the optical density. Absorbance was taken at 570 nm by Infinite 200PRO reader (Tecan, Männedorf, Switzerland) [18,25].

### 4.8. Heamolytic Assay

Fresh human blood was collected from the healthy volunteer’s antecubital (the informed consent form signed from the volunteer’s) for the heamolytic assay. This study was approved by the ethics committee of the University Hospital Brno (Brno, Czech Republic) according to MZ ČR čj. MZDZ 39683/2012, date: 6 November 2012.

At first, the blood samples were mixed with 150 mM NaCl and centrifuged at 2000× g for 5 min to remove plasma and serum and this was repeated thrice. In the second step, red blood cells (RBC) were diluted with 7.4 pH PBS and henceforth RU-S4 complex was added in different concentrations (25 µg/mL and 50 µg/mL). Triton X-100 0.1% and PBS were used as positive and negative controls. The samples were mixed gently and incubated for 1 h at 37 °C. After the incubation period, the optical density of the samples was taken at λ = 540 nm followed by the centrifugation at 3000× g for 10 min. Hemolysis percentage was calculated by this following equation:

% hemolysis = 100 × ((Abs of samples − Abs of Neg.control) ÷ (Abs of Pos.control − Abs of Neg.control))(1)

where the absorbance of the supernatant obtained from the samples incubated with Ruthenium complex denotes Abs of samples; and the negative control, named Abs of negative control obtained from the absorbance of the supernatant of the PBS pH 7.4 incubated sample; and A100% is the 0.1% Triton X-100 incubated samples supernatant absorbance (complete lysis).

### 4.9. Optical Microscopy and Fluorescence Microscopy Imaging of RU-S4 Treated Bacteria

Bacterial samples were incubated with RU-S4 for LIVE/DEAD cell imaging. Bacterial live/dead cell evaluation was done by optical Fluorescence microscopy technique. Two different kinds of fluorescence dye were used for labeling bacterial strains. SYTO 9 dye (Thermo Fischer Scientific, cat# S34854) was used to stain live cells and propidium iodide fluorescent dye to detect live/dead both cells. Inverted Olympus IX 71S8F-3 fluorescence microscope (Olympus Corporation, Tokyo, Japan) was used which was equipped with Olympus UIS2 series objective LUCplanFLN 40X (N.A. 0.6, WD 2.7-4mm, F.N. 22) and a mercury arc lamp X-cite 12 (120 W; Lumen Dynamics, Mississauga, ON, Canada) [8,18]. Four different kind of bacterial samples were used in this experiments VRSA (CCM 1767) MRSA (ST239: SCCmecIIIA), *S. aureus* (NCTC 8511) and hospital sample *Staphylococcus epidermidis*.

### 4.10. Electron Microscopy Imaging of Bacteria with RU-S4 Compound

Cryo-SEM using a plunge freezing method was used for imaging and the samples were stored in liquid nitrogen [28]. Cryo-SEM visualization of samples was performed with FEI Versa3D equipped with a Quorum cryo stage and transfer station (FEI Company).

### 4.11. In vivo Infection Model Preparation

For in vivo infection model preparation and treatment, eight weeks old female Bulb/c mice were used [6]. Three different set of animals were prepared. Each set contained 5 bulb/c mice (Table 1). The mice were anesthetized by intramuscular injection of ketamine (Narkamon^®^, Spofa a.s., Prague, Czech Republic) at 100 mg/kg and xylazine (Rometar^®^, Spofa a.s., Prague, Czech Republic) at 10 mg/kg with an insulin syringe [47,48]. Mice fur was removed by the Nair^®^ hair remover lotion (Church and Dwight Co. Inc., Princeton, NJ, USA) and electric trimmer as described previously [49]. The mice were shaved one day before the infection introduction experiment [38]. The mice infection model prepared by subcutaneous injection of 0.05 mL of 1 × 107 live VRSA (CCM 1767) culture [39,49,50,51,52]. All animal management and experiments were performed with the approval of the Ethics Commission at the Faculty of AgriSciences, Mendel University in Brno, Czech Republic in accordance with Act No. 246/1992 Coll. on the protection of animals against cruelty. Throughout the experiment, atmospheric conditions were maintained at 22 ± 1 °C, 60% humidity, and the light administration (12 h L, 12 h D) with a maximum illumination of 200 lx.

### 4.12. In Vivo Infection Treatment

Infected mice were treated with Ru-S4. The Compound was applied (50 µg/mL concentration) by spray and subcutaneous injection, two times in a day until 15 days according to our experimental setup [53,54,55,56]. During the application the RU-S4 final concentration was 50 µg/mL. Images of all three set of mice were taken by a Nikon DSLR camera in every 24 h interval.

### 4.13. In Vivo Imaging

Near-infrared (NIR) fluorescent agent as imaging probes are very useful for high-affinity imaging. Matrix metalloproteinase-based fluorescence dye MMP Sense 645 Fast (Perkin Elmer cat# NEV10100) was used for in vivo mice imaging, detection of the infection state and for the depletion of the infection level and cure with time. Fluorescent dye was reconstituted with sterile 1.2 mL PBS and made it to 4 nmol from 48 nmol. Each mouse received 100 µL of the solution through tail vein injection with a 1 mL insulin syringe (BD). This experiment was repeated five times and the images were taken by Carestream In Vivo Xtreme with light source 400 W Xenon bulb [40,57,58,59].

## Figures and Tables

**Table 1:** Animal set preparation.

Animal Set	Purpose
First set	Infected model with drug administration
Second set	Infected model without drug administration
Third set	Uninfected control

## Supplementary Materials

The following are available online at https://www.mdpi.com/1422-0067/21/7/2656/s1, Figure S1: (a) SEM and (b) EDS elemental mapping. (c–h) stands for chloride, ruthenium, very less amount of carbon, negligible presence of sulphur, high amount of nitrogen, and less amount of oxygen; Figure S2: EDS spectrum where presence of ruthenium with high concentration of nitrogen can be seen; Figure S3: Growth curve of RuCl3 treated VRSA (CCM 1767); Figure S4: Growth curve of RuCl3 treated MRSA (ST239: SCCmecIIIA); Figure S5: Growth curve of 2-(1H-benzimidazol-2-ylmethylsulfanylmethyl)-1H-benzimidazole treatment against VRSA (CCM 1767); Figure S6: Growth curve of 2-(1H-benzimidazol-2-ylmethylsulfanylmethyl)-1H-benzimidazole treatment against MRSA (ST239: SCCmecIIIA); Figure S7: Growth curve of Schiff base 2- [(E)-1H-1,2,4-triazol-5-yliminomethyl] phenol treatment against VRSA (CCM 1767); Figure S8: Growth curve of Schiff base 2-((E)-1H-1,2,4-triazol-5-yliminomethyl) phenol treatment against MRSA (ST239: SCCmecIIIA); Figure S9 (a) cytotoxicity for RU-S4 against the human cell line; (b) PNT1A untreated and treated cells; (c) MTT assay of PNT1A cell line treated with RU-S4.; Figure S10: Healthy animal without any infection and treatment; Figure S11: Infected mice without drug administration. (a) after bacterial infection dose, (b) day 1 Infection initiation, (c) day 3 Infection and wound growth, (d) day 6 inflammation and swelling, with growing region internally as well; Figure S12: MMP Sense fluorescence intensity decreased day by day due to the treatment and cure of infection wound; Figure S13: Optical microscopy image of *S. epidermidis*; Figure S14: LIVE/DEAD cell imaging of *S. epidermidis*; Supplementary experimental setup 1, 1.1 Toxicological (LD100) studies in Bulb/c mice, 1.2. LD50 calculation, 1.3. Result.
