# Supplementary material for: A Novel Ruthenium Based Coordination Compound Against Pathogenic Bacteria
Source: Int J Mol Sci. 2020 Apr 10;21(7):2656. doi: 10.3390/ijms21072656 (PMC7178087; doi:10.3390/ijms21072656)
Supplement: Supplementary file 1 [file ijms-21-02656-s001.pdf]

International Journal of
Molecular Sciences

MDPI

Supplementary

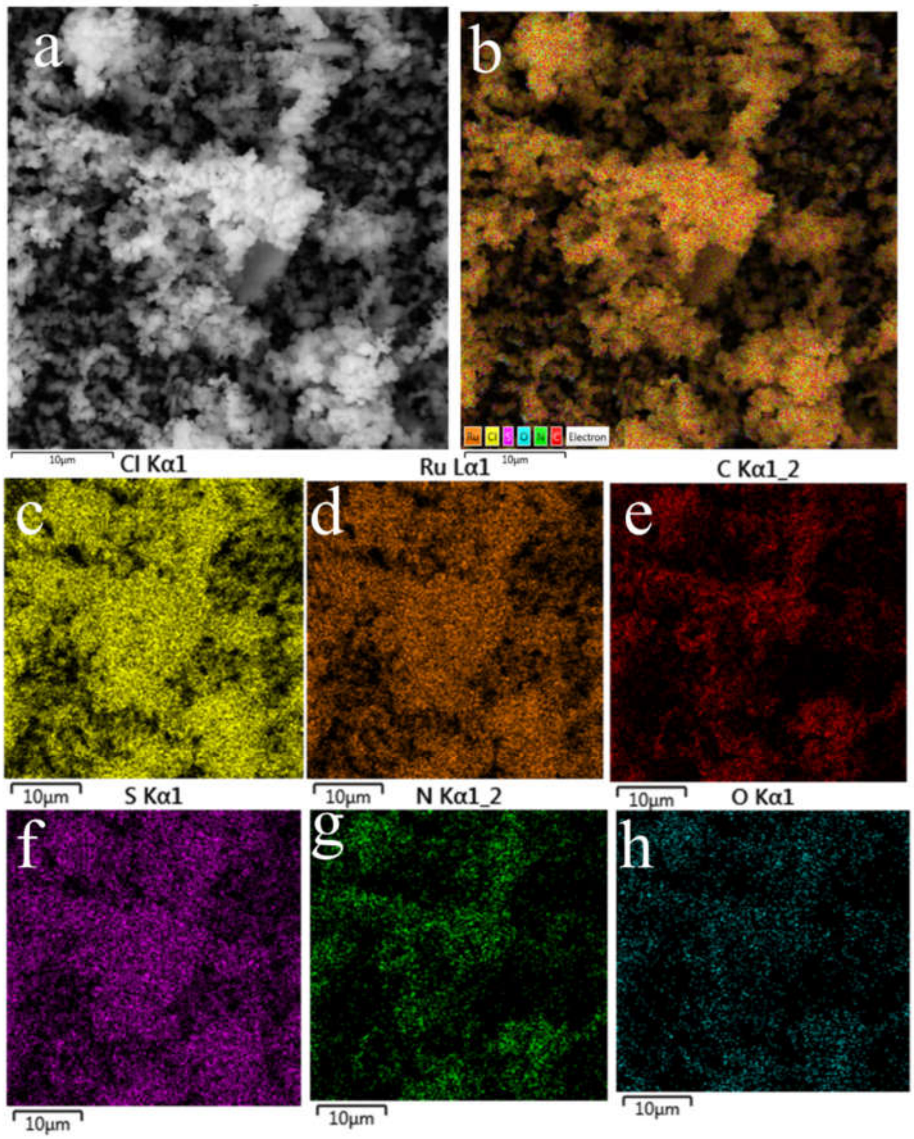

Figure S1. (**a**) SEM image of RU-S4; (**b**) EDS elemental mapping of RU-S4 (merged image of c,d,e,f,g,h); (**c**) elemental imaging of Ru-S4 and presence of chloride; (**d**) elemental imaging of Ru-S4 and presence ruthenium; (**e**) elemental imaging of Ru-S4 and presence of carbon; (**f**) elemental imaging of Ru-S4 and presence of sulfur; (**g**) elemental imaging of Ru-S4 and presence of nitrogen; (**h**) elemental imaging of Ru-S4 and presence of oxygen.

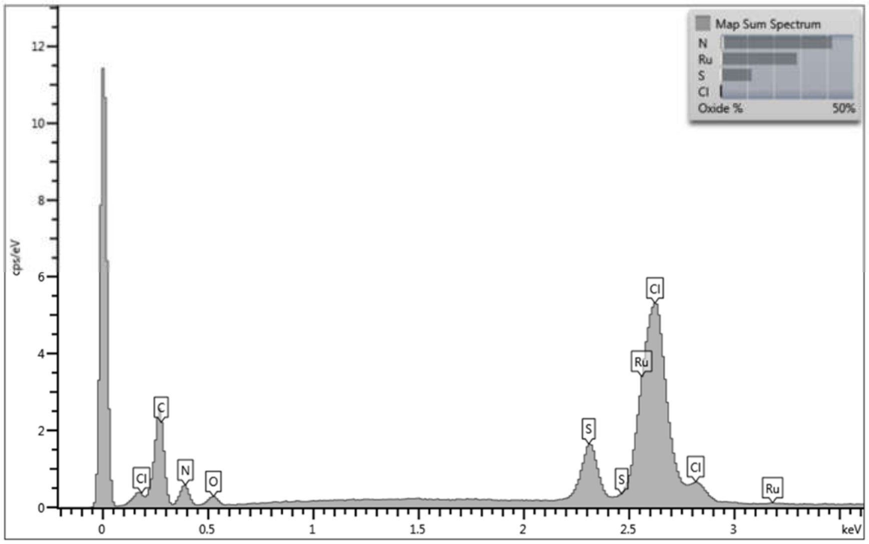

Figure S2. EDS spectrum where presence of ruthenium with high concentration of nitrogen can be seen.

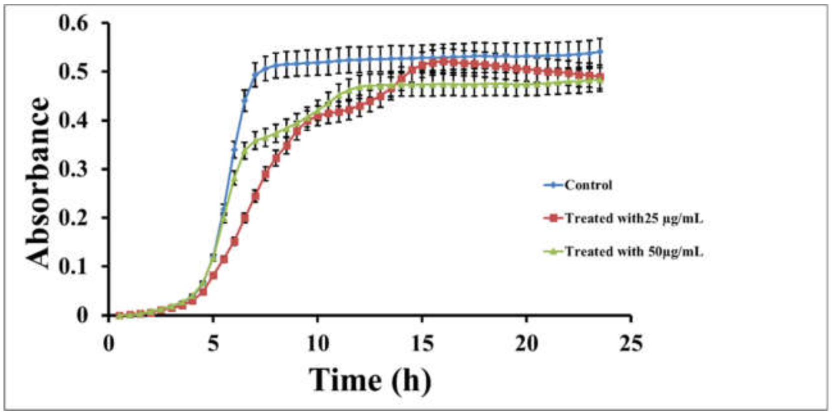

Figure S3. Growth curve of $RuCl_3$ treated VRSA. Data represent the mean ±SD, $n = 3$.

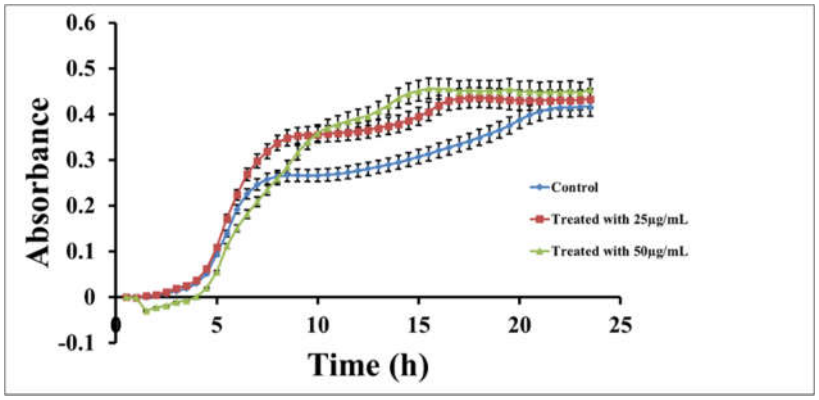

Figure S4. Growth curve of $RuCl_3$ treated MRSA. Data represent the mean ±SD, $n = 3$.

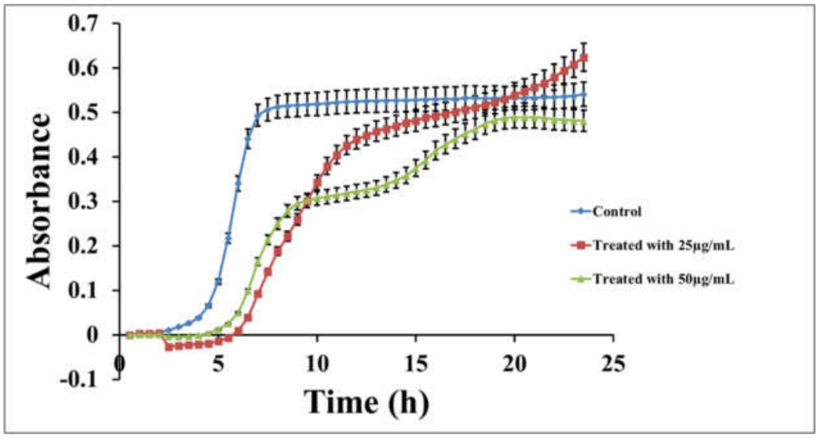

Figure S5. Growth curve of 2-(1H-benzimidazol-2-ylmethylsulfanylmethyl)-1H-benzimidazole treatment against VRSA. Data represent the mean ±SD, *n* = 3.

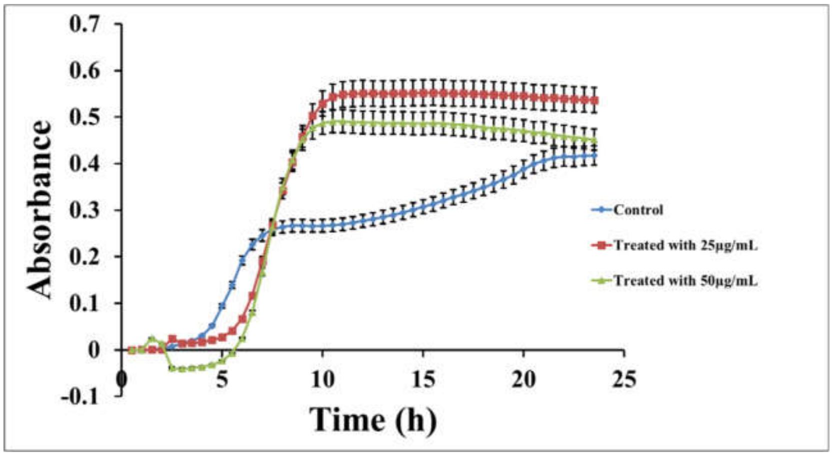

Figure S6. Growth curve of 2-(1H-benzimidazol-2-ylmethylsulfanylmethyl)-1H-benzimidazole treatment against MRSA. Data represent the mean ±SD, *n* = 3.

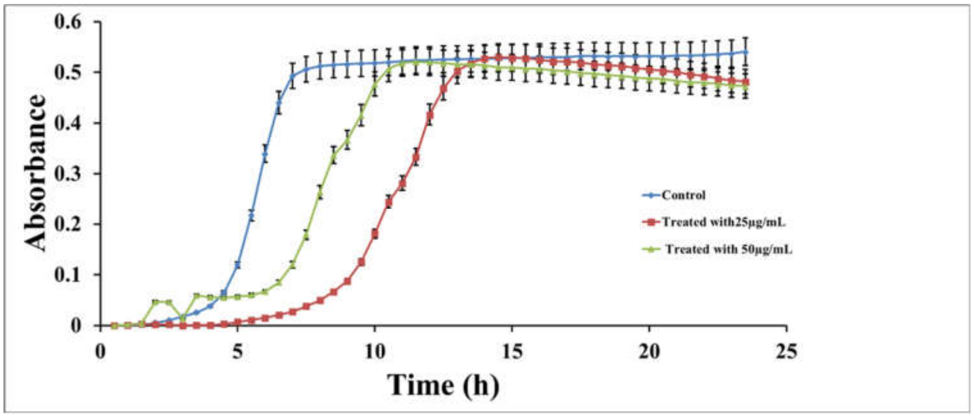

Figure S7. Growth curve of Schiff base 2-[(E)-1H-1,2,4-triazol-5-yliminomethyl-phenol treatment against VRSA. Data represent the mean ±SD, *n* = 3.

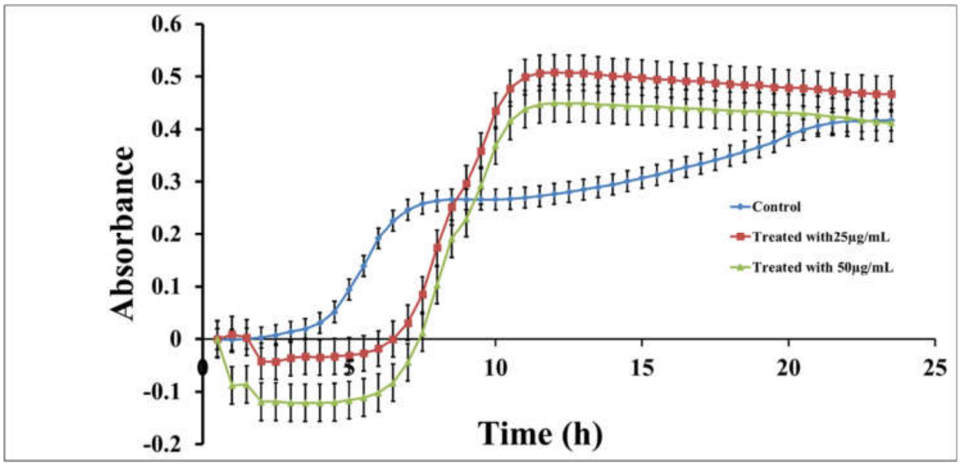

Figure S8. Growth curve of Schiff base 2-[(E)-1H-1,2,4-triazol-5-yliminomethyl] phenol treatment against MRSA. Data represent the mean ±SD, $n = 3$.

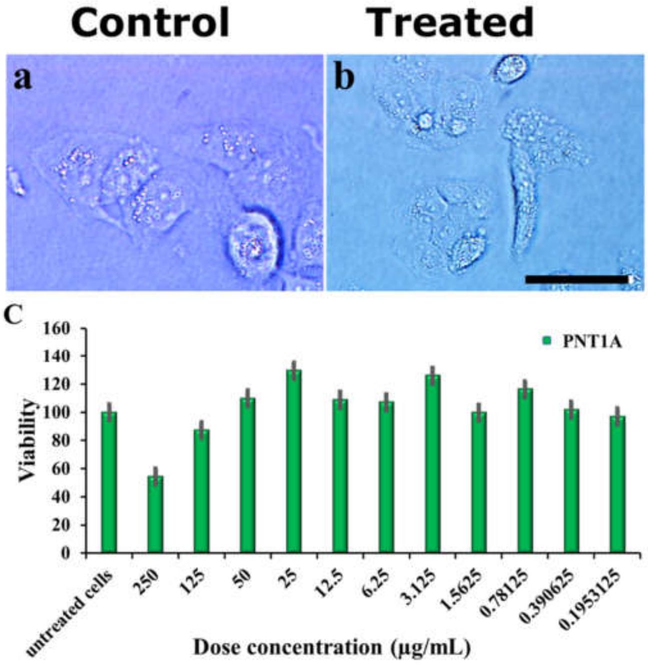

Figure S9. (**a**) cytotoxicity for RU-S4 against the human cell line; (**b**) PNT1A untreated and treated cells; (**c**) MTT assay of PNT1A cell line treated with RU-S4.

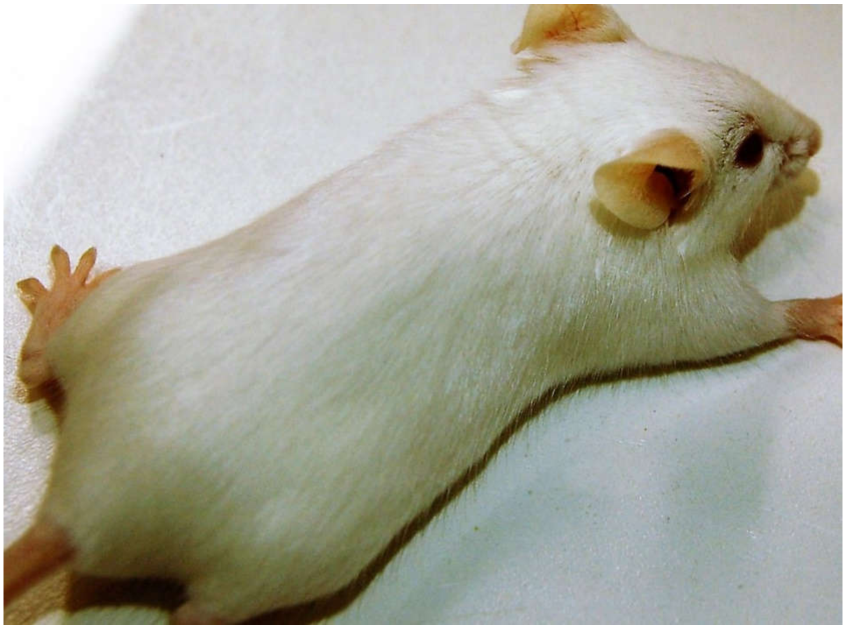

Figure S10. Healthy animal without any infection and treatment.

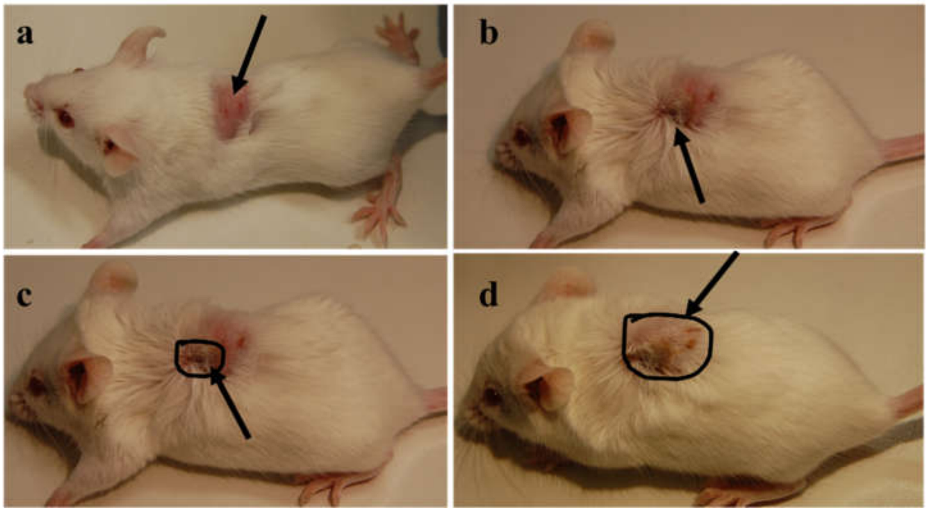

Figure S11. Infected mice without drug administration. (**a**) after bacterial infection dose; (**b**) day 1 infection initiation; (**c**) day 3 Infection and wound growth; (**d**) day 6 inflammation and swelling, with growing region internally as well.

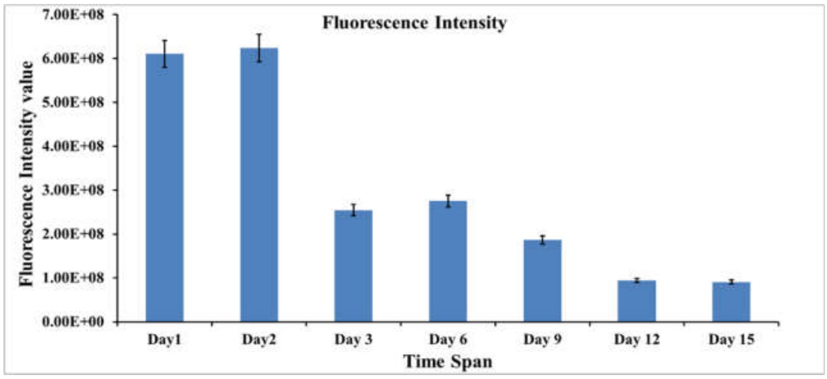

Figure S12. MMP Sense fluorescence intensity decreased day by day due to the treatment and cure of infection wound.

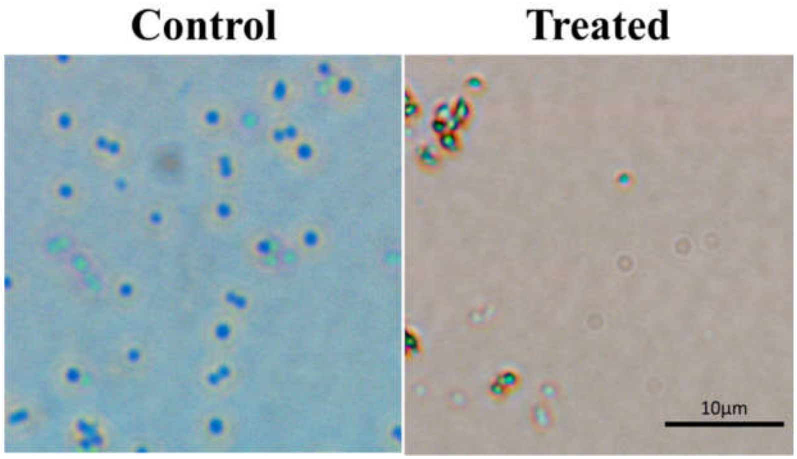

Figure S13. Optical microscopy image of *S. epidermidis*.

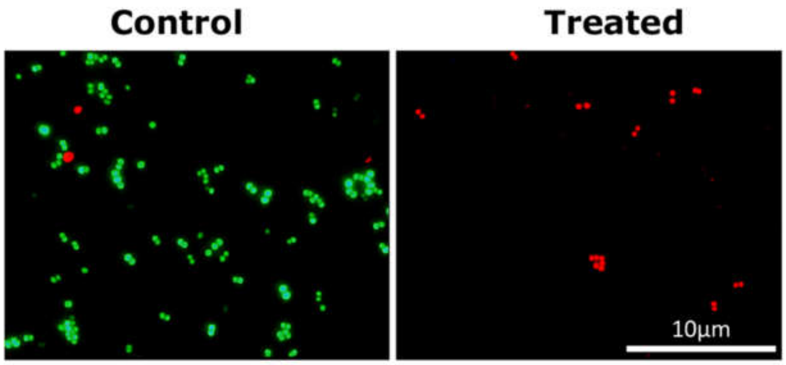

Figure S14. LIVE/DEAD cell imaging of *S. epidermidis*.

1. Supplementary Experimental Section

1.1. Toxicological (LD_{100}) Studies in Bulb/c Mice

Acute toxicity of a drug can be determined by the calculation of LD50. The dose that will kill 50% of animals of a particular species. LD_{50} is a very important experimental setup for drug molecule administration and dose determination [1,2].

Five groups containing 5 Bulb/c mice (22-26 g) in each were used in this study. All animals were treated Intraperitoneal (I.P.) [3,4] (Table 1). All animals weight was measure before the RU-S4 dose administration. All the animals were kept under continuous observation for 12 hours after the administration of dose, for any change in behavior or physical activities. After 24 h, all survived mice were anesthetized by intramuscular injection of ketamine (Narkamon®, Spofa a.s., Prague, Czech Republic) at 100 mg/kg and xylazine (Rometar®, Spofa a.s., Prague, Czech Republic) at 10 mg/kg with an insulin syringe and harvested in accordance with Act No. 246/1992 Coll. on the protection of animals against cruelty, Czech Republic. Throughout the experiment, microclimatic conditions were maintained at 23 ± 1 °C, 60% humidity, and the light regime (12 h L, 12 h D) with a maximum illumination of 200 lx [5].

1.2. LD_{50} Calculation

LD100= Lethal dose causing the 100% death of all test animals.
a = the difference between two successive doses of administered extract/substance
b = the average number of dead animals in two successive doses.
n = total number of animals in group
Karber (1931) arithmetic method was used for the determination of LD 50.

Table S1. Animal grouping for Toxicological studies (LD_{50}).

Groups	No of Animals	Dose	Route of Administration
Control	5	PBS (10 mL/kg)	I.P.
Treated Group I	5	RU-S4 (10 mg/kg)	I.P.
Treated Group II	5	RU-S4 (50 mg/kg)	I.P.
Treated Group III	5	RU-S4 (100 mg/kg)	I.P.
Treated Group IV	5	RU-S4 (200 mg/kg)	I.P.

1.3. Results

This experimental observation and calculation reveal that the synthesized RU-S4 toxic with LD_{100} at 200 mg/kg body weight of experimental animals as in the first 5 hours of observation 100% morbidity was observed. As per observations and calculations the LD_{50} value of synthesized RU-S4 after i.p. administration was found 160 mg/kg body weight. After the dose application no morbidity were observed in the treated group I and treated group II, whereas in treated group IV 100% morbidity was observed and in treated group III 60% morbidity was found (Table S2). Hodge and Sterner toxicity scale was used for the toxicity level evaluation (Table S3).

Table S2. Dose toxicity and animal mortality.

Groups	Mortality of Animals (x/n)	Dose	Route of Administration
Control	0/5	PBS (5 ml/kg)	I.P.
Treated Group I	0/5	RU-S4 (10 mg/kg)	I.P.
Treated Group II	0/5	RU-S4 (50 mg/kg)	I.P.
Treated Group III	3/5	RU-S4 (100 mg/kg)	I.P.
Treated Group IV	5/5	RU-S4 (200 mg/kg)	I.P.

Table S3. Hodge and Sterner Toxicity Scale.

Toxicity Level and Term	LD50
Extremely Toxic	Less than 1 mg/Kg
Highly Toxic	1–50 mg/Kg
Moderately Toxic	50–500 mg/Kg
Slightly Toxic	500–5000 mg/Kg
Practically Non-Toxic	5000–15000 mg/Kg

References

1. Chaudhary, M.; Srivastava, A.; Ali, S. Acute Toxicity (Lethal Dose 50 Calculation): Medicine as a life saver or destroyer. *International Journal* **2019**, *7*, 216-218.
2. Chen, Z.; Meng, H.; Xing, G.; Chen, C.; Zhao, Y.; Jia, G.; Wang, T.; Yuan, H.; Ye, C.; Zhao, F., et al. Acute toxicological effects of copper nanoparticles in vivo. *Toxicology Letters* **2006**, *163*, 109-120, doi:https://doi.org/10.1016/j.toxlet.2005.10.003.
3. Domingo, J.L. ALUMINUM (ALUMINIUM) | Toxicology. In *Encyclopedia of Food Sciences and Nutrition (Second Edition)*, Caballero, B., Ed. Academic Press: Oxford, 2003; https://doi.org/10.1016/B0-12-227055-X/00035-3pp. 160-166.
4. Ahmed, M.; Azmat, A. Acute toxicity (lethal dose 50 calculation) and histopathological effects of methanolic extract of Berberis vulgaris in mice. *World J. Pharmacol. Res.* **2014**, *3*, 1439-1448.
5. Horky, P.; Skalickova, S.; Urbankova, L.; Baholet, D.; Kociova, S.; Bytesnikova, Z.; Kabourkova, E.; Lackova, Z.; Cernei, N.; Gagic, M., et al. Zinc phosphate-based nanoparticles as a novel antibacterial agent: in vivo study on rats after dietary exposure. *Journal of Animal Science and Biotechnology* **2019**, *10*, 17, doi:10.1186/s40104-019-0319-8.

© 2020 by the authors. Submitted for possible open access publication under the terms and conditions of the Creative Commons Attribution (CC BY) license (http://creativecommons.org/licenses/by/4.0/).
